# Clinicopathologic characteristics, diagnostic clues, and prognoses of patients with multiple sporadic gastrointestinal stromal tumors: a case series and review of the literature

**DOI:** 10.1186/s13000-020-00939-7

**Published:** 2020-05-14

**Authors:** Yan-Ying Shen, Xin-Li Ma, Lin-Xi Yang, Wen-Yi Zhao, Lin Tu, Chun Zhuang, Bo Ni, Qiang Liu, Ming Wang, Hui Cao

**Affiliations:** 1 Department of Gastrointestinal Surgery, Renji Hospital, School of Medicine, Shanghai Jiaotong University, 160 Pujian Road, Shanghai, 200127 People’s Republic of China; 2 Department of Pathology, Renji Hospital, School of Medicine, Shanghai Jiaotong University, Shanghai, People’s Republic of China

**Keywords:** Gastrointestinal stromal tumor, Multiple, Sporadic, KIT mutations, PDGFRA mutations

## Abstract

**Background:**

Most sporadic gastrointestinal stromal tumors (GISTs) occur as solitary tumors, while multiple sporadic GISTs are extremely rare and often misdiagnosed as metastatic GISTs, leading to inappropriate treatment. This study aimed to investigate the clinicopathological characteristics, diagnostic clues, and prognoses of multiple sporadic GISTs.

**Methods:**

Twenty-seven patients with multiple sporadic GISTs and 11 patients with metastatic GISTs mimicking sporadic GISTs were analyzed. The clinicopathological characteristics, genetic mutation types, and prognoses were summarized. In addition, 1066 cases of primary GISTs with a single lesion diagnosed at the same hospital were included as controls.

**Results:**

Compared with 1066 cases of primary GIST with a single lesion, multiple sporadic GISTs occurred at an older age, were more common in women than in men, and were located mainly in the stomach. They were generally small in size, had a low mitotic index and were more often rated as very low risk/low risk. Mutation analysis of all available lesions revealed different KIT/PDGFRA mutation patterns among tumors from the same patients. No patient relapsed during the follow-up period. Among 11 patients with metastatic GISTs that mimicked multiple sporadic GISTs, multiple lesions from the same patient always had concordant pathological and mutational characteristics; namely, they carried an identical KIT/PDGFRA mutation, and the mitotic index was usually high.

**Conclusions:**

The prognoses of patients with multiple sporadic GISTs were not worse than those of patients with a single lesion of the same risk under the same treatment. When it was difficult to distinguish multiple sporadic GISTs from metastatic GISTs, multiple lesions in the same patient carried different KIT/PDGFRA mutation patterns, which supported tumor multiplicity, while the concordant hypermitotic phase in multiple lesions of GISTs suggested that the tumor was metastatic.

## Background

Gastrointestinal stromal tumors (GISTs) are the most common gastrointestinal, mesenchymal neoplasm [1]. Activating KIT or PDGFRA mutations represent early initiating molecular events in a majority of GISTs [1]. GISTs are clinically heterogeneous, ranging from clinically benign, intermediate-risk tumors to frank sarcomas [2, 3]. GISTs of different risks require different clinical management strategies.

GISTs can arise from any part of the GI tract, predominantly in the stomach and small intestine and rarely in extragastrointestinal locations [2]. Most GISTs occur as solitary tumors. In contrast, familial GISTs (patients with germline KIT or PDGFRA mutations) and special subtypes of GISTs (for example, neurofibromatosis type 1 (NF1)-related GISTs, sporadic succinate dehydrogenase (SDH)-deficient GISTs, and Carney triad syndrome-related GISTs) usually develop as multiple lesions [4–7]. However, GISTs originating multifocally in patients lacking evidence of familial or syndromic GISTs or SDH-deficient GISTs which were mentioned above rarely exist. Sometimes it is difficult to distinguish multiple sporadic GISTs from metastatic GISTs. The clinical management of these patients is not mentioned in current guidelines since reports of the clinicopathological features and prognostic information of multiple sporadic GISTs are still very limited [8–14].

In this study, we examined 27 patients with multiple sporadic GISTs without evidence of familial or syndromic GISTs or SDH-deficient GISTs. The clinicopathological characteristics, genetic mutation types, and prognoses were summarized. These features were compared with those of primary GISTs with a single lesion or metastatic GISTs in our center and with the case series reported in the previous literature. This study will enable us to provide evidence for the proper clinical management of multiple sporadic GISTs in the future.

## Methods

### Patient selection

**Fig. 1:**
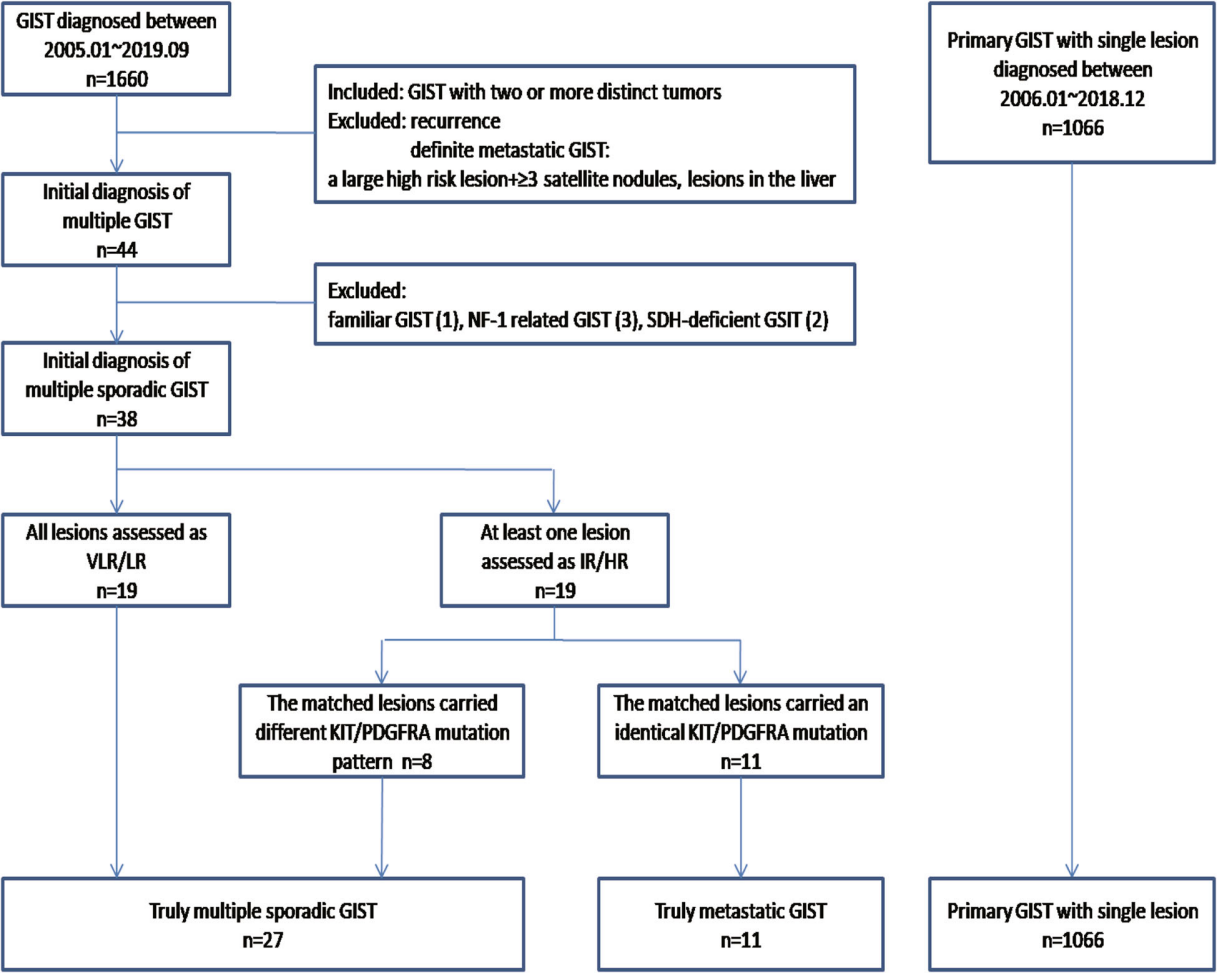
Patient selection in this study

We reviewed the preoperative imaging data, surgical records, and histopathological records of all 1660 patients diagnosed with GISTs between January 2005 and September 2019 at Renji Hospital, School of Medicine, Shanghai Jiaotong University, Shanghai, China. Follow-up information was obtained during regular outpatient visits or by telephone calls to the patients. Patient selection is shown in Fig. 1. Initially, a total of 44 patients with two or more distinct GIST lesions without definite evidence of recurrence and metastasis were included. Definite evidence of metastasis was defined as a large, high-risk main tumor lesion with three or more small satellite nodules or lesions in the liver. Three patients with multiple intestinal GISTs had a known NF1 history. One patient had a familial GIST. The KIT K642E mutation was detected in six tumor lesions and blood samples from this patient. Two patients had SDH-deficient GISTs without a history of Carney triad syndrome. These six patients had special subtypes of GISTs with their own unique clinical characteristics and were excluded from further analysis.

The remaining 38 patients with multiple GISTs were the subjects of this study. In 19 patients in whom all lesions were assessed as very low or low risk, the lesions were separated from each other grossly and then directly diagnosed as multiple GISTs (Table 1, cases 1–19). The other 19 patients all had at least one lesion that was assessed as intermediate or high risk. Since there was some doubt as to whether this group of patients had multiple GISTs or metastatic GISTs, we performed genetic testing for each lesion. A different KIT/PDGFRA mutation pattern was detected in 8 cases, supporting an independent origin for the matched lesion. These 8 cases were diagnosed as multiple sporadic GISTs (Table 1, cases 20–27). In the other 11 cases, the matched lesions carried an identical KIT/PDGFRA mutation, suggesting a clonal relationship. No KIT/PDGFRA gene mutation was detected in normal tissues around the tumors of these 11 patients, which could rule out the possibility that they were familial GISTs. We evaluated the prognosis of the 11 patients, and the lesions were reclassified as metastatic GISTs (Table 2). Hence, truly multiple, sporadic, and nonspecific subtypes of GISTs occurred in 27 cases. In addition, 1066 cases of primary GISTs with a single lesion between January 2006 and December 2018 were also included as the control. This study was approved by the Ethics Committee of Renji Hospital, School of Medicine, Shanghai Jiao Tong University.

**Table 1:** Clinicopathologic and molecular features of 27 patients with multiple sporadic GISTs

No	Age/Sex	Site/size (cm)/MF/Risk	Histotype	Mutation status	Imatinib/follow-up/months
1a	60/F	ST/0.8/≤5/VLR	Spindle	NA	No/NED/120
1b		ST/2/≤5/VLR	Spindle	NA	
2a	71/F	ST/1/≤5/VLR	Spindle	NA	No/NED/108
2b		ST/2/≤5/VLR	Spindle	NA	
3a	75/F	ST/0.4/≤5/VLR	Spindle	NA	No/Died of GC/34
3b		ST/0.5/≤5/VLR	Spindle	NA	
4a	54/F	ST/1/≤5/VLR	Spindle	NA	No/NED/104
4b		ST/1.2/≤5/VLR	Mix	NA	
5a	62/F	ST/1/≤5/VLR	Spindle	NA	No/NED/102
5b		ST/1/≤5/VLR	Spindle	NA	
6a	47/F	ST/3.5/< 5/LR	Spindle	KIT: p.V560del	No/NED/86
6b		ST/2/≤5/VLR	Spindle	KIT: p.V559D	
7a	63/M	ST/2/≤5/VLR	Spindle	KIT: p.V559A	No/NED/60
7b		ST/0.5/≤5/VLR	Spindle	KIT: p.K642E	
8a	65/F	ST/1.5/≤5/VLR	Epithelioid	PDGFRA: p.D842V	No/NED/58
8b		ST/1/≤5/VLR	Spindle	KIT: p.L576P	
9a	61/M	ST/3.5/≤5/LR	Spindle	KIT: p.W557G	No/NED/54
9b		ST/1.5/≤5/VLR	Spindle	Wild type	
10a	71/F	ST/3.5/≤5/LR	Spindle	KIT: p.L576P	No/NED/12
10b		ST/0.5/≤5/VLR	Spindle	KIT: p.V559G	
11a	68/F	ST/1/≤5/VLR	Spindle	KIT: p.W557_E561del	No/NED/43
11b		ST/0.8/≤5/VLR	Spindle	KIT: p.V559_V560del	
12a	70/F	ST/3.8/≤5/LR	Spindle	KIT: p.W557R	No/NED/34
12b		ST/1/≤5/VLR	Spindle	KIT: p.V560D	
13a	72/F	SI/1.2/≤5/VLR	Spindle	KIT: p.L576P	No/Died of CC/34
13b		SI/0.8/≤5/VLR	Spindle	KIT p.D579del	
14a	65/M	ST/4/≤5/LR	Mix	NA	No/NED/148
14b		ST/1/≤5/VLR	Mix	NA	
15a	77/F	ST/5/≤5/LR	Mix	PDGFRA: p.D842V	No/NED/18
15b		ST/2/≤5/VLR	Spindle	KIT: p.N822K	
16a	67/M	ST/5/≤5/LR	Spindle	KIT: p.V560del	No/NED/6
16b		ST/0.7/≤5/VLR	Spindle	KIT: p.L576P	
17a	74/F	ST/4/≤5/LR	Spindle	KIT: p.W557_K558del	No/NED/4
17b		ST/1/≤5/VLR	Spindle	Wild type	
17c		ST/0.3/≤5/VLR	Spindle	NA	
18a	70/F	ST/1.8/≤5/VLR	Spindle	KIT: p.W557R	No/NED/12
18b		ST/1/≤5/VLR	Spindle	KIT: p.D820Y	
18c		ST/0.7/≤5/VLR	Spindle	KIT: p.Q556_I563del	
19a	71/M	ES/2.5/≤5/LR	Spindle	KIT: p.P577_H580dup	No/DUNC/25
19b		ES/1.5/≤5/VLR	Spindle	KIT: p.W557_V559indelF	
20a	58/F	ST/6/≤5/IR	Spindle	KIT: p.Y578_H580dup	Yes/NED/52
20b		ST/8/≤5/IR	Spindle	KIT: p.W558_E561del	
21a	76/F	ST/2.5/≤5/LR	Spindle	KIT: p.P577_H580dup	No/NED/32
21b		ST/4.5/6–10/IR	Spindle	KIT: p.M552_W557del	
22a	69/M	ST/7/≤5/IR	Spindle	KIT: p.W557R	Yes/NED/10
22b		ST/3.5/≤5/LR	Spindle	KIT: p.V560E	
23a	63/M	ST/6.5/≤5/IR	Spindle	KIT: p.P551_M552del	Yes/NED/48
23b		ST/5.2/≤5/IR	Spindle	KIT: p.I571_D579dup	
24a	65/M	SI/6.5/≤5/HR	Spindle	KIT: p.G565_L576del	Yes/NED/8
24b		SI/5.5/≤5/HR	Spindle	KIT: p.A502_Y503dup	
24c		SI/1.2/≤5/VLR	Spindle	KIT: p.W557R	
25a	70/F	ST/13/6–10/HR	Spindle	KIT: p.L576_D579dup	Yes/NED/5
25b		ST/1.5/≤5/VLR	Epithelioid	PDGFRA: p.D842Y	
26a	61/F	SI/7/≤5/HR	Spindle	KIT: p.N566_T574del	Yes/NED/62
26b		SI/5/≤5/LR	Spindle	KIT: p.V560E	
^a^27a	71/M	ST/2/≤5/VLR	Spindle	KIT: p.W557_K558del	No/NED/42
^a^27b		ST/1/≤5/VLR	Spindle	KIT: p.K642E	
^a^27c		ST/6/> 10/HR	Spindle	KIT:p.A502_Y503dup	Yes/NED/18

Abbreviation: *MF*, Mitotic count per 50 high power field, *ST* Stomach, *SI* Small intestine, *ES* Esophagus, *Risk* Risk assessment according to modified NIH scheme, *VLR* Very low risk, *LR* Low risk, *IR* Intermediate risk, *HR* High risk, *NA* Not available, *NED* No evidence of disease, *DUNC* Died of unknown cause, *GC* Gastric cancer, *CC* Colon cancer

^a^Case 27: The patient had metachronous GISTs. He had two GIST lesions in the stomach initially. Then, he developed another GIST in the stomach 42 months after the first surgery. We did not think it was a recurrence because the genotype of this tumor was different from that of the previous two lesions

**Table 2:** Clinicopathologic and molecular features of 11 patients with metastatic GISTs mimicking multiple sporadic GISTs

No	Age/Sex	Site/size (cm)/MF/Risk	Histotype	Mutation status	Imatinib/ follow-up/months
1a	43/M	SI/6.5/> 10/HR	Spindle	KIT: p.W557_K558del	Yes/NED, 12
1b		SI/3.5/> 10/HR	Spindle	KIT: p.W557_K558del	
1c		SI/1/> 10/HR	Spindle	KIT: p.W557_K558del	
2a	67/F	ST/14/≤5/HR	Spindle	KIT: p.W557_K558indelE	Yes/AWD/37
2b		ST/1/≤5/VLR	Spindle	KIT: p.W557_K558indelE	
3a	65/M	SI/11.5/≤5/HR	Spindle	KIT: p.A502_Y503dup	Yes/NED/35
3b		SI/6.5/≤5/HR	Spindle	KIT: p.A502_Y503dup	
4a	66/F	ST/4.5/> 10/HR	Spindle	KIT: p.K558_V559del	Yes/DOD/68
4b		ST/3/> 10/HR	Spindle	KIT: p.K558_V559del	
5a	69/M	SI/9/≤5/HR	Spindle	KIT: p.V559D	Yes/AWD/89
5b		SI/6/≤5/HR	Spindle	KIT: p.V559D	
6a	73/M	ST/5/> 10/HR	Spindle	KIT: p.V555_V559del	Yes/NED/16
6b		ST/3.5/> 10/HR	Spindle	KIT: p.V555_V559del	
7a	39/F	VA/2.5/> 10/HR	Spindle	KIT: p. K558del	Yes/AWD/12
7b		VA/2.5/> 10/HR	Spindle	KIT: p. K558del	
8a	56/F	ME/3.5/6–10/HR	Spindle	KIT: p.A502_Y503dup	Yes/AWD/46
8b		ME/1.5/6–10/IR	Spindle	KIT: p.A502_Y503dup	
8c		ME/1/6–10/IR	Spindle	KIT: p.A502_Y503dup	
9a	65/M	SI/11/> 10/HR	Spindle	KIT: p.Y553_K558del	No/DOD/18
9b		SI/9/> 10/HR	Spindle	KIT: p.Y553_K558del	
10a	62/M	ST/13/> 10/HR	Spindle	KIT: p. K558_V559del	Yes/NED/6
10b		ST/5/> 10/HR	Spindle	KIT: p. K558_V559del	
11a	70/M	ST/17/> 10/HR	Spindle	KIT: p.M552_V560del	Yes/AWD/38
11b		ST/3/> 10/HR	Spindle	KIT: p.M552_V560del	

Abbreviation: *MF* Mitotic count per 50 high power field, *ST* Stomach, *SI* Small intestine, *VA* Vagina; *ME* Mesentery, *Risk* Risk assessment according to modified NIH scheme, *VLR* Very low risk, *LR* Low risk, *IR* Intermediate risk, *HR* High risk, *NA* Not available, *NED* No evidence of disease, *AWD* Alive with disease, *DOD* Died of disease

### Histopathological evaluation

The histopathological diagnosis of a GIST was confirmed by two pathologists before the patients were included in the study. The largest tumor diameter and mitotic count per 50 high-power field (HPF) were evaluated as recommended by the international criteria for each tumor lesion. Risk stratification was performed according to the modified NIH scheme [3].

### Mutation analysis

Mutation analysis was performed with genomic DNA isolated from formalin-fixed paraffin-embedded tissues by using macrodissection and standard procedures. KIT exons 9, 11, 13, and 17 and PDGFRA exons 12 and 18 were amplified. The amplification products were purified and subjected to bidirectional Sanger sequencing by using an ABI 3130 Sequencer (Applied Biosystems, Inc., Foster City, CA).

### Statistical analysis

Frequency tables were analyzed using the χ^2^ test. Disease-free survival (DFS) was defined as the time between the date of surgery and the date of relapse. Survival analysis was conducted using the Kaplan–Meier method, and survival between groups was compared by using the log-rank test. All reported *p* values were two-tailed, and statistical significance was defined as *p* < 0.05. Analyses were performed using Statistical Package for the Social Sciences (SPSS) version 16.0 (SPSS, Inc., Chicago, IL, USA).

## Results

### Incidence of multiple sporadic GISTs

After excluding familial GISTs (*n* = 1), syndromic GISTs (*n* = 3, all with NF-1), SDH-deficient GISTs (*n* = 2), and metastatic GISTs that mimicked multiple GISTs (*n* = 11), 27 cases were identified as multiple sporadic GISTs, representing 1.63% of 1660 consecutive surgically resected GISTs in our hospital.

### Clinicopathologic characteristics

The clinicopathological features of all 27 patients with multiple sporadic GISTs are summarized in Table 1. There were 9 males and 18 females. The age at diagnosis ranged from 47 to 77 years (median age, 68 years). All patients had lesions located in an identical organ. Among all 58 tumors from 27 patients, the most common tumor site was the stomach (*n* = 49, 84.5%), followed by the small intestine (*n* = 7, 12.1%), and only two tumors from the same patient were located in the esophagus (3.4%). The tumor diameter ranged from 0.3 to 13 cm. Of these tumors, 35 (60.3%) were ≤ 2 cm, 13 (22.4%) were > 2 cm and ≤ 5 cm, 9 (15.5%) were > 5 cm and ≤ 10 cm, and only 1 tumor (1.7%) was > 10 cm. The mitotic count per 50 high-power field (HPF) was ≤5 in 55 (94.8%) GISTs, 6–10 in 2 (3.4%) GISTs, and > 10 in 1 GIST. Based on cell morphology, the tumors were classified as spindle (52/58, 89.7%), mixed (4/58, 6.9%), or epithelioid (2/58, 3.4%). According to the modified NIH scheme, the risk of recurrence was very low in 35 (60.3%) tumors, low in 12 (20.7%) tumors, intermediate in 6 (10.3%) tumors, and high in only 5 (8.6%) tumors. Most patients had two lesions, and only three patients had three lesions. Compared with primary GISTs with only one lesion diagnosed at our center between January 2006 and December 2018 (Table 3), multiple sporadic GISTs occurred at an older age, were more common in women than in men, and were located mainly in the stomach. They were generally small in size, had a low mitotic index and were more often rated as very low risk/low risk. These differences were significant (all *p* < 0.05).

**Table 3:** Comparison of clinicopathological features between multiple sporadic GISTs and primary GISTs with only one lesion

		Multiple sporadic GISTs (58 tumors from 27 patients)	Primary GIST with one lesion (1066 tumors from 1066 patients)	*p*
Age (years)	≤60	14.8%, 4/27	48.8%, 520/1066	< 0.001
	> 60	85.2%, 23/27	51.2%, 546/1066	
Sex	Male	33.3%, 9/27	53.7%, 572/1066	0.037
	Female	66.7%, 18/27	46.3%, 494/1066	
location	Stomach	84.5%, 49/58	60.3%, 643/1066	< 0.001
	Non-stomach	15.5%, 9/58	39.7%, 423/1066	
Tumor size	≤2 cm	60.3%, 35/58	10.7%, 114/1066	< 0.001
	2-5 cm	22.4%, 13/58	42.8%, 456/1066	
	5-10 cm	15.5%, 9/58	34.1%, 363/1066	
	> 10 cm	1.7%, 1/58	12.5%, 133/1066	
Mitosis	≤5/50HPF	94.8%, 55/58	75.7%, 807/1066	0.002
	6–10/50HPF	3.4%, 2/58	12.9%, 137/1066	
	> 10/50HPF	1.7%, 1/58	11.4%, 122/1066	
Risk assessment (modified NIH)	VLR	60.3%, 35/58	9.4%, 100/1066	< 0.001
	LR	20.7%, 12/58	38.2%, 407/1066	
	IR	10.3%, 6/58	17.5%, 187/1066	
	HR	8.6%, 5/58	34.9%, 372/1066	

Abbreviation: *HPF* high power field, *VLR* very low risk, *LR* low risk, *IR* intermediate risk, *HR*, high risk

**Fig. 2:**
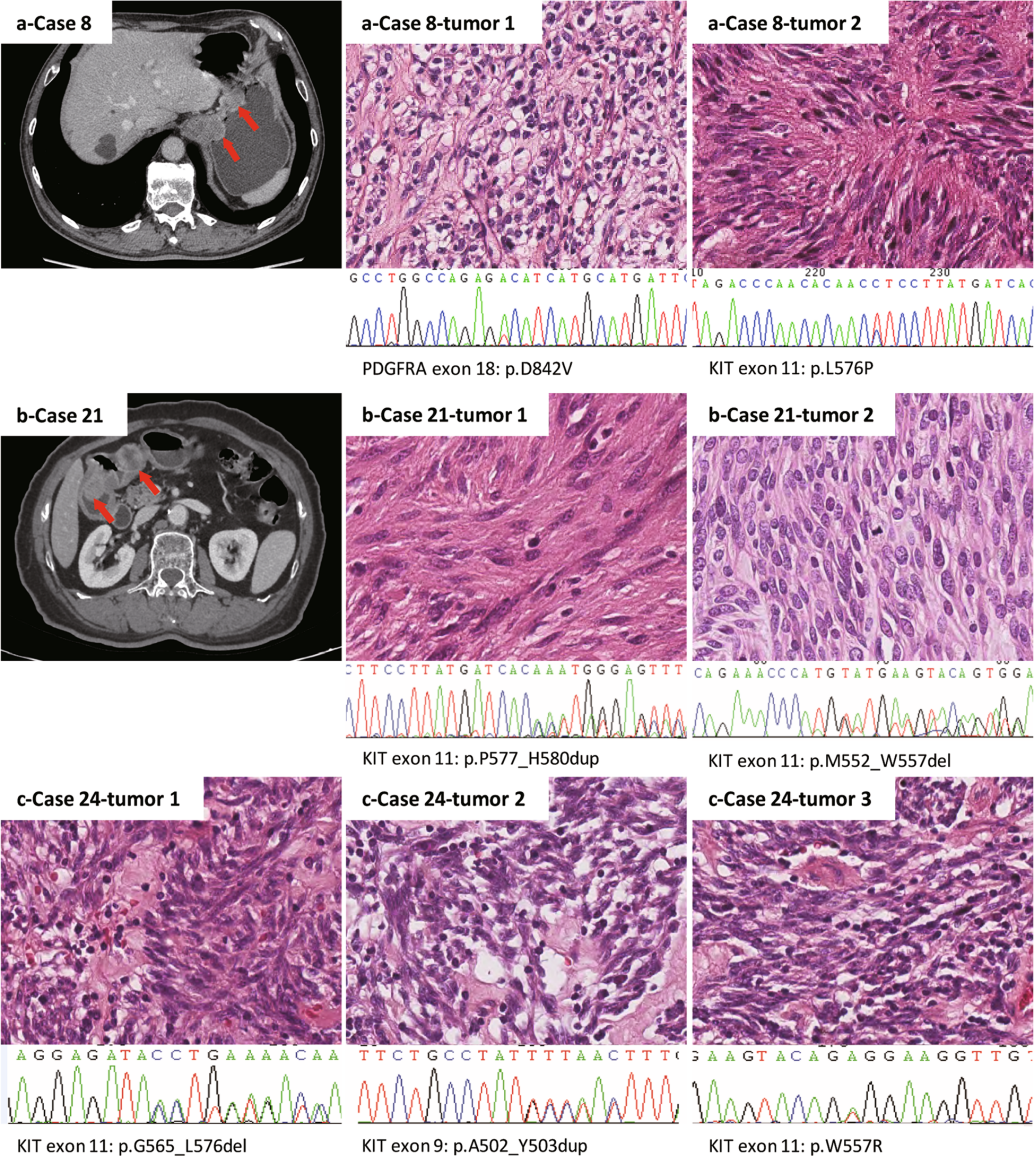
**a** A CT scan shows (red arrow) that there are two distinct GIST lesions in the stomach (case 8 in Table 1). The histological features and genetic types of the two lesions were different. The tumor cells in the first tumor were epithelioid, with the PDGFRA D842V mutation, while those in the second tumor were spindle shaped, with the KIT L576P mutation. **b** A CT scan shows (red arrow) two lesions of the same GIST patient (case 21 in Table 1). Although the tumor cells were spindle shaped, the cell density, atypia, mitosis and genetic types of the two lesions were different. **c** shows no difference in histological features among the three lesions from the same patient (case 24 in Table 1), but the genotypes of the three lesions were not the same

We also compared the clinicopathologic characteristics of different tumors in the same patient. In 20 cases, the paired tumors were comparable in size; in the other seven cases, a major lesion at least three times as large as the other lesion was identified. Paired tumors displayed concordant morphology (spindle/spindle; mixed/mixed) in the majority of cases (85.2%, 23/27) and discordant morphology (spindle/mixed; spindle/epithelioid) in four cases (Fig. 2). The mitotic index of the paired lesions was concordantly low in 25 cases, whereas a different mitotic index was observed in three cases (Fig. 2). The risk assessment of the paired lesions was concordantly very low, low, intermediate and high in 10, 0, 2, and 0 cases, respectively, whereas a different risk assessment was observed in 15 cases.

### Molecular findings

Mutation analysis was successfully performed in a total of 45 tumors from 21 patients with multiple sporadic GISTs. KIT mutations in exons 9, 11, 13, and 17 were detected in 2, 34, 2, and 2 lesions, respectively. Three lesions had PDGFRA mutations, and two lesions were wild-type. Among all cases of multiple sporadic GISTs in our study, different mutations were detected among tumors from the same patient.

### Prognostic information of patients with multiple sporadic GISTs

Among 27 patients with multiple sporadic GISTs, all GIST lesions were assessed as very low or low risk in 19 patients. None of 19 patients received imatinib therapy after surgery. During a median follow-up period of 48 months (range: 4–148 months), none of 19 patients relapsed. Three patients died of other diseases, and 17 patients remained alive. The recurrence rate of multiple sporadic GISTs (all lesions were assessed as very low risk/low risk) tended to be lower than that of primary GISTs with a single lesion of the same risk diagnosed at our center (of 487 patients with single, very-low-risk and low-risk GISTs, 10 patients relapsed). However, the difference was not significant (*p* = 0.448).

Among the other eight patients with multiple sporadic GISTs (at least one lesion was assessed as intermediate risk or high risk), seven patients received imatinib therapy (three patients with an intermediate-risk tumor for 1 year and four patients with a high-risk tumor for 3 years) after surgery and only one patient who had two intermediate-risk lesions did not. They also were alive with no evidence of GIST recurrence. However, among 177 cases of intermediate-risk GIST with a single lesion in our center, 69 patients received imatinib for 1 year, and 108 patients did not; two and three patients, respectively, relapsed. Among 348 cases of high-risk GIST (most of whom received imatinib therapy for 3 or more years) with a single lesion in our center, 122 cases recurred. In the group of intermediate- or high-risk GISTs, the prognoses of patients with multiple sporadic GISTs were also not worse than those of patients with a single lesion of the same risk under the same treatment. No statistical analysis was performed because the number of patients with multiple sporadic GISTs (at least one lesion was assessed as intermediate risk or high risk) was too small.

### Comparative analysis of clinicopathological characteristics and genetic types of multiple sporadic GISTs with at least one lesion assessed as intermediate or high risk and metastatic GISTs mimicking multiple sporadic GISTs

In our study, it was initially difficult to determine whether 11 cases were multiple or metastatic GISTs because the genetic analysis of KIT/PDGFRA failed to show tumor multiplicity. Because of the diverse KIT and PDGFRA gene mutations in GISTs, the probability of two or more tumors from the same patient carrying the same genetic type was very small. The multiple lesions from the same patient with very-low-risk or low-risk GISTs (which were not considered metastatic GISTs) always had different KIT/PDGFRA mutation types, which also confirmed this hypothesis. In addition, the 11 patients had a very poor prognosis (all patients but one received imatinib therapy). During the follow-up period, five patients relapsed, two died of GISTs, and four remained alive without disease. Hence, we regarded these 11 cases as metastatic GISTs.

**Fig. 3:**
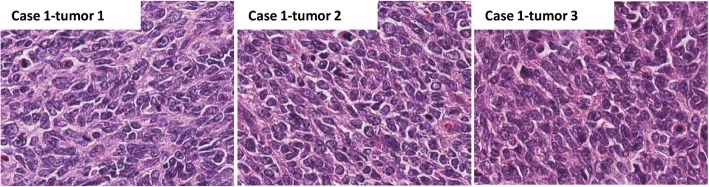
Shows that the three lesions from this GIST patient had concordance, high tumor cell density and obvious atypia. The mitotic index was more than 10/50 HPF. The three lesions had the same KIT mutation pattern. Finally, this case was considered a metastatic GIST

Among all 18 lesions from 7 patients in the truly multiple GIST group (at least one lesion assessed as intermediate or high risk), the tumor diameter ranged from 1 to 13 cm, with a median diameter of 5.35 cm. The mitotic index of most lesions (83.3%, 15/18) was less than 5/50 HPF. Among all 24 lesions from 11 patients in the truly metastatic GIST group, the tumor diameter ranged from 1 to 17 cm, with a median diameter of 4 cm. The mitotic count was ≤5 in 6 (25%) GISTs, 6–10 in 3 (12.5%) GISTs, and > 10 in 15 (62.5%) GISTs (Fig. 3). Moreover, the mitotic index was always concordant in the multiple lesions from the same patients. A concordant hypermitotic phase in multiple lesions suggested that the tumor was metastatic.

## Discussion

Multiple sporadic GISTs are very rare, and previous reports have been mostly individual cases or have included only a small number of cases. In the current study, 27 cases of multiple sporadic GISTs were identified among 1660 cases of GISTs diagnosed at our center by histological features and an accurate genotype analysis. The incidence of multiple sporadic GISTs was 1.6%. To our knowledge, this is the largest report of multiple sporadic GISTs to date. It is generally believed that multiple GISTs are common in familial GISTs or syndrome-related GISTs, such as NF-1- or Carney triad syndrome-related GISTs. However, to our surprise, most of the multiple GISTs were sporadic without evidence of familial GISTs, syndromic GISTs or SDH-deficient GISTs in our series. This result was similar to that from another study conducted in China [14]. Among 20 patients with multiple GISTs in Li el al.’s study, 2 patients had NF-1-related GISTs, 2 patients had Carney triad syndrome-related GISTs, and 16 patients had sporadic GISTs.

Multiple sporadic GISTs reported in our study usually occurred at an old age, were more common in females than in males and were located mainly in the stomach. They were generally small in size, had a low mitotic index and were more often rated as very low risk/low risk. These features were in accordance with the results of previous studies except for the predilection for females [9–14]. The results of the study by Kang et al. and ours showed that multiple sporadic GISTs were more common in females than in males [10], while the results of the studies by Li et al., Haller et al., and Agaimy et al. showed that they were slightly more prevalent in males than in females [9, 12, 14]. This discrepancy might be due to bias caused by the small sample size.

For the first time, we performed a stratified survival analysis of the prognosis of multiple sporadic GISTs. Both our study and previous studies have shown that patients with multiple sporadic GISTs (all lesions assessed as very low risk/low risk) had a very favorable prognosis. No patient, including the 19 patients in our study and the 28 patients in the literature, relapsed during follow-up [9–14]. For multiple sporadic GISTs with at least one lesion assessed as intermediate or high risk, none of eight patients (all patients but one received imatinib therapy after surgery) relapsed in our study. We also reviewed the prognoses of these patients in the previous literature. Of 22 multiple sporadic GIST patients with at least one lesion assessed as intermediate and high risk mentioned in the literature [9–14], 13 cases showed tumor multiplicity on KIT/PDGFRA mutation analysis, excluding them as metastatic GISTs. Among these 13 patients, four and nine patients had intermediate- and high-risk lesions, respectively. None of the patients with intermediate-risk lesions received imatinib therapy or relapsed. Of the 9 patients with high-risk lesions, three patients received imatinib therapy, and 6 patients did not, and one patient and 3 patients relapsed, respectively. In general, the prognoses of patients with multiple sporadic GISTs were not worse than those of patients with a single lesion at the same risk under the same treatment.

In our study, multiple lesions from the same patients with very-low-risk or low-risk GISTs (which were not considered metastatic GISTs) always had different KIT/PDGFRA mutation types. This result was similar to that of previous studies [9–14]. Among the 23 cases of multiple sporadic GISTs (very low or low risk) reported in the previous literature, only 3 cases had identical genotypes in different lesions from the same patient. We analyzed the details of these 3 patients. In Agaimy et al.’s study, the patient had two GIST lesions that were not very far apart but were separated by a thin capsule [13]. Whether this case was a truly multiple GIST was questionable. One case of multiple sporadic GISTs with wild-type lesions for KIT/PDGFRA mutation analysis had been reported [13]. (In the group of multiple sporadic GISTs with intermediate or high risk lesions, there were also three cases showed wild-type lesions). This situation did not exist in our study, which may be due to the exclusion of SDH-deficient GISTs. Patients with this particular subtype of GISTs were often wild-type for KIT/PDGFRA genes and had multiple tumors. This was a recently recognized subtype of GISTs that had not been identified in the previous literature. Only one case with a very low risk multiple sporadic GIST showed an identical KIT point mutation in Kang et al.’s study [10]. Both the results of our study and those of previous reports demonstrate the fact that the probability of two or more tumors from the same patient carrying the same genetic type was very small.

For those patients with multifocal GISTs (at least one lesion evaluated as intermediate or high risk), if the KIT/PDGFRA mutation pattern of paired lesions is different, there is no doubt that they are multiple sporadic GISTs, and if the paired lesions have an accordant genotype, whether these patients have multiple sporadic GISTs or metastatic GISTs remains controversial. In the study by Gasparotto et al., three such cases were reported [11]. Two patients with follow-up information received imatinib therapy, and one relapsed. The author regarded these cases as “truly metastatic GISTs”. In contrast, such cases were classified as multiple sporadic GISTs in the studies by Li et al. (1 case) and Kang et al. (3 cases) [10, 14]. In Li et al.’s study, the patient had two high-risk GIST lesions and died of GISTs during the follow-up even with imatinib. In Kang et al.’s study, two patients had at least an intermediate-risk GIST lesion, and one patient had a high-risk GIST lesion. They did not receive imatinib and had no relapse. Similar to the studies by Gasparotto et al. and Li et al., the 11 patients with multifocal GISTs (paired lesions with the same KIT/PDGFRA mutation pattern) had a very poor prognosis in our study. Five patients relapsed, and two died of GISTs during the follow-up period. Therefore, we reclassified these patients as metastatic GIST patients. Although more comprehensive genetic testing was still needed to confirm whether they had metastatic GISTs, it was at least necessary to distinguish these lesions from truly multiple sporadic GISTs (with tumor multiplicity confirmed by a different KIT/PDGFRA mutation pattern), since their prognoses were different. Namely, when there are intermediate or high-risk lesions in multiple sporadic GISTs and their genotypes are consistent, the possibility of metastatic GISTs should be considered. In addition to the KIT/PDGFRA mutation pattern, our results also showed that histological features such as a concordant hypermitotic phase in multiple lesions could also help distinguish multiple GISTs from metastatic GISTs. Agaimy et al. reported that the involvement of the muscularis propria was a potentially reliable marker for primary GISTs [12].

## Conclusions

In summary, multiple sporadic GISTs were rare. The prognoses of patients with multiple sporadic GISTs were not worse than those of patients with a single lesion at the same risk under the same treatment. Multiple tumor lesions did not increase the risk of tumor recurrence. KIT/PDGFRA gene mutation analysis was necessary for each multifocal GIST lesion and could help us distinguish between multiple GISTs and metastatic GISTs. In addition, a concordant hypermitotic phase in multiple GIST lesions suggested that the tumor was metastatic.

## Data Availability

There are no additional supporting data available.

## Abbreviations

AWD: Alive with disease
CC: Colonic cancer
DOD: Died of disease
DUNC: Died of unknown cause
ES: Esophagus
GC: Gastric cancer
GIST: Gastrointestinal stromal tumors
HPF: High power field
HR: High risk
IR: Intermediate risk
LR: Low risk
ME: Mesentery
MF: Mitotic count per 50 high power field
NA: Not available
NED: No evidence of disease
NF1: Neurofibromatosis type 1
SDHB: Succinate dehydrogenase
SI: Small intestine
ST: Stomach
VA: Vagina
VLR: Very low risk

## References

[1] Corless CL, Barnett CM, Heinrich MC (2011). Gastrointestinal stromal tumours: originand molecular oncology. Nat Rev Cancer.

[2] Miettinen M, Lasota J (2006). Gastrointestinal stromal tumors: pathology and prognosis at different sites. Semin Diagn Pathol.

[3] Joensuu H (2008). Risk stratification of patients diagnosed with gastrointestinal stromal tumor. Hum Pathol.

[4] Neuhann TM, Mansmann V, Merkelbach-Bruse S, Klink B, Hellinger A, Hoffkes HG (2013). A novel germline KIT mutation (p.L576P) in a family presenting with juvenile onset of multiple gastrointestinal stromal tumors, skin hyperpigmentations, and esophageal stenosis. Am J Surg Pathol.

[5] Miettinen M, Fetsch JF, Sobin LH, Lasota J (2006). Gastrointestinal stromal tumors in patients with neurofibromatosis 1: a clinicopathologic and molecular genetic study of 45 cases. Am J Surg Pathol.

[6] Miettinen M, Wang ZF, Sarlomo-Rikala M, Osuch C, Rutkowski P, Lasota J (2011). Succinate dehydrogenase -deficient GISTs: a clinicopathologic, immunohistochemical, and molecular genetic study of 66 gastric GISTs with predilection to young age. Am J Surg Pathol.

[7] Matyakhina L, Bei TA, McWhinney SR, Pasini B, Cameron S, Gunawan B (2007). Genetics of carney triad: recurrent losses at chromosome 1 but lack of germline mutations in genes associated with paragangliomas and gastrointestinal stromal tumors. J Clin Endocrinol Metab.

[8] Li J, Ye Y, Wang J, Zhang B, Qin S, Shi Y (2017). Chinese consensus guidelines for diagnosis and management of gastrointestinal stromal tumor. Chin J Cancer Res.

[9] Haller F, Schulten HJ, Armbrust T, Langer C, Gunawan B, Fuzesi L (2007). Multicentric sporadic gastrointestinal stromal tumors (GISTs) of the stomach with distinct clonal origin: differential diagnosis to familial and syndromal GIST variants and peritoneal metastasis. Am J Surg Pathol.

[10] Kang DY, Park CK, Choi JS, Jin SY, Kim HJ, Joo M (2007). Multiple gastrointestinal stromal tumors: Clinicopathologic and genetic analysis of 12 patients. Am J Surg Pathol.

[11] Gasparotto D, Rossi S, Bearzi I, Doglioni C, Marzotto A, Hornick JL (2008). Multiple primary sporadic gastrointestinal stromal tumors in the adult: an underestimated entity. Clin Cancer Res.

[12] Agaimy A, Markl B, Arnholdt H, Wunsch PH, Terracciano LM, Dirnhofer S (2009). Multiple sporadic gastrointestinal stromal tumours arising at different gastrointestinal sites: pattern of involvement of the muscularis propria as a clue to independent primary GISTs. Virchows Arch.

[13] Agaimy A, Dirnhofer S, Wunsch PH, Terracciano LM, Tornillo L, Bihl MP (2008). Multiple sporadic gastrointestinal stromal tumors (GISTs) of the proximal stomach are caused by different somatic KIT mutations suggesting a field effect. Am J Surg Pathol.

[14] Li K, Tjhoi W, Shou C, Yang W, Zhang Q, Liu X (2019). Multiple gastrointestinal stromal tumors: analysis of clinicopathologic characteristics and prognosis of 20 patients. Cancer Manag Res.

